# Supplementary material for: Deep mutational scanning quantifies DNA binding and predicts clinical outcomes of PAX6 variants
Source: Mol Syst Biol. 2024 Jun 7;20(7):825–44. doi: 10.1038/s44320-024-00043-8 (PMC11219921; doi:10.1038/s44320-024-00043-8)
Supplement: Supplementary file 1 — Appendix [file 44320_2024_43_MOESM1_ESM.pdf]

Appendix

Table of Contents

Appendix Figures S1-S5……………………………………………….…………………….pages 2-6

Appendix Tables S1-S3………………………………………………..……………….…..pages 7-13

Appendix References……………………………………………….……………………..pages 14-17

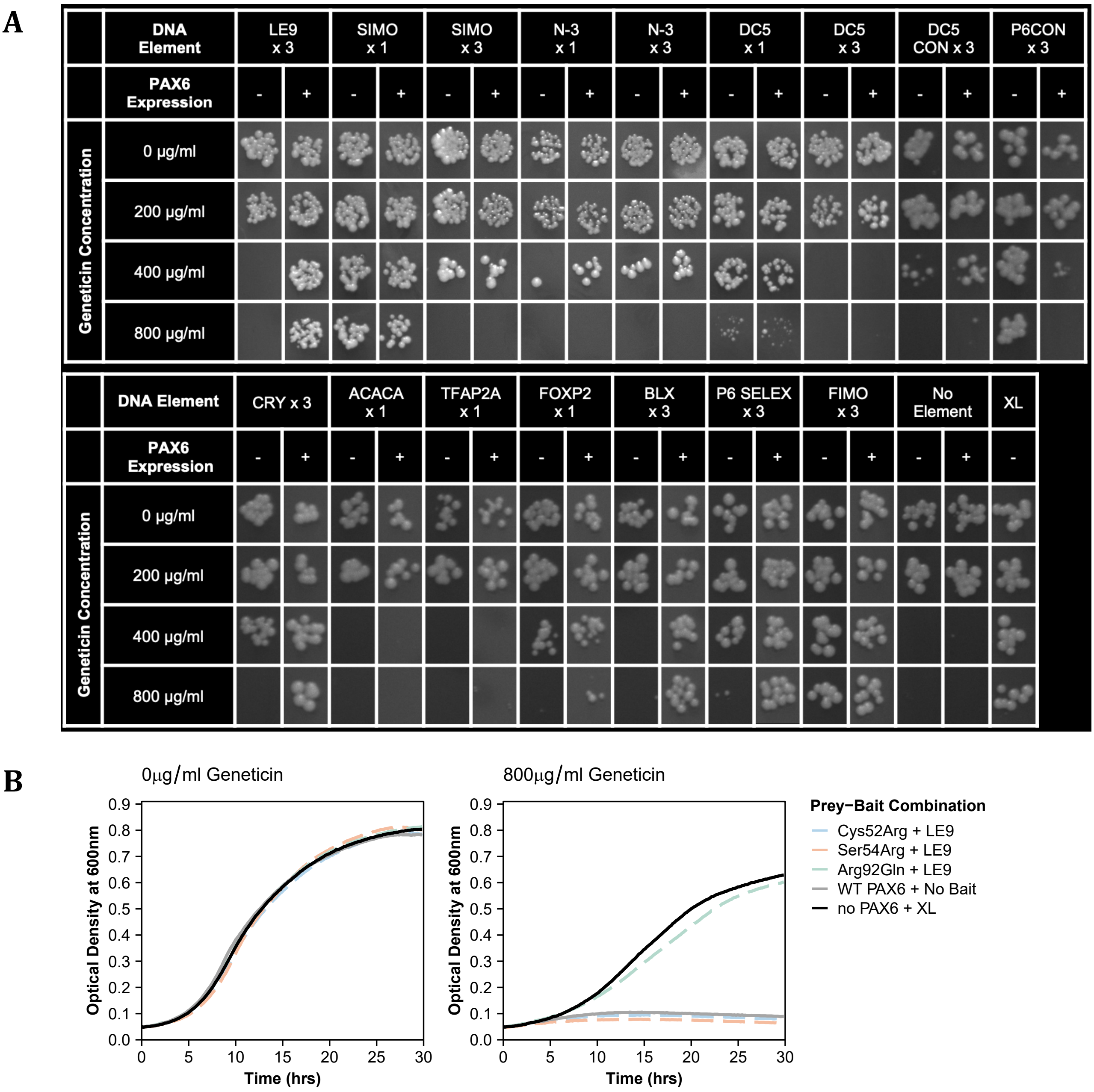

Appendix Figure S1. Bait-prey screening and validation of growth assay

(a) Spot assays used to screen PAX6:bait combinations. Strains with single (x1) or three tandem repeats (x3) of PAX6 bait sequences, a synthetic minimal yeast promoter (XL), and a strain lacking a bait sequence (No Element) are shown. Each PAX6 bait-specific strain was screened with (+) and without (-) PAX6 expression over a range of geneticin concentrations. (b) Liquid culture validation of the LE9 x3 bait strain using characterised PAX6 variants. Three PAX6 variants with varying levels of reported disruption to DNA-binding (Williamson *et al*, 2019) are represented as dashed lines. Variants Cys52Arg (aniridia) and Ser54Arg (non-aniridia) showed substantially perturbed binding to LE9 in the previous study, while Arg92Gln (benign) showed milder disruption to LE9 binding. Log growth rate was measured by optical density.

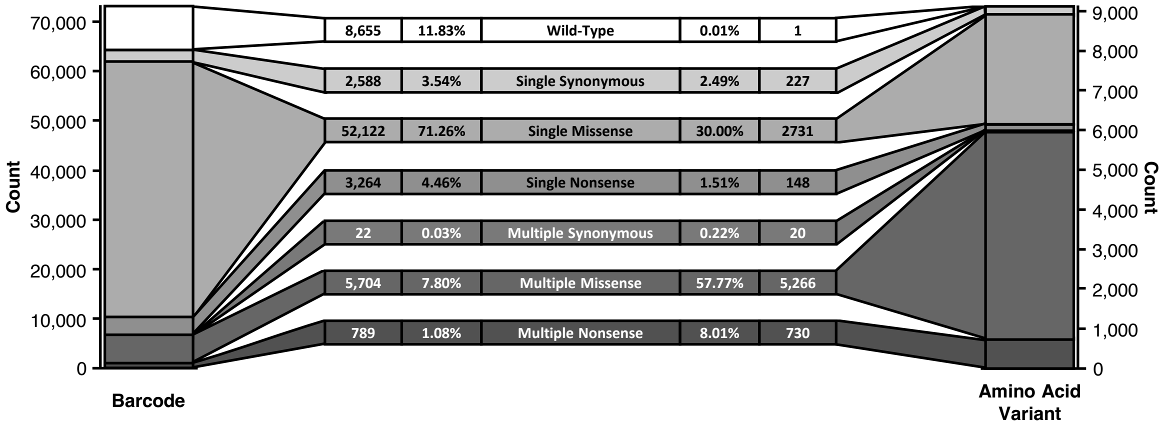

Appendix Figure S2. Variant library composition

(a) Library composition following saturation mutagenesis broken down by unique barcodes (left) and unique variants (right). The number of each variant by type and corresponding percentage of the total of each are shown.

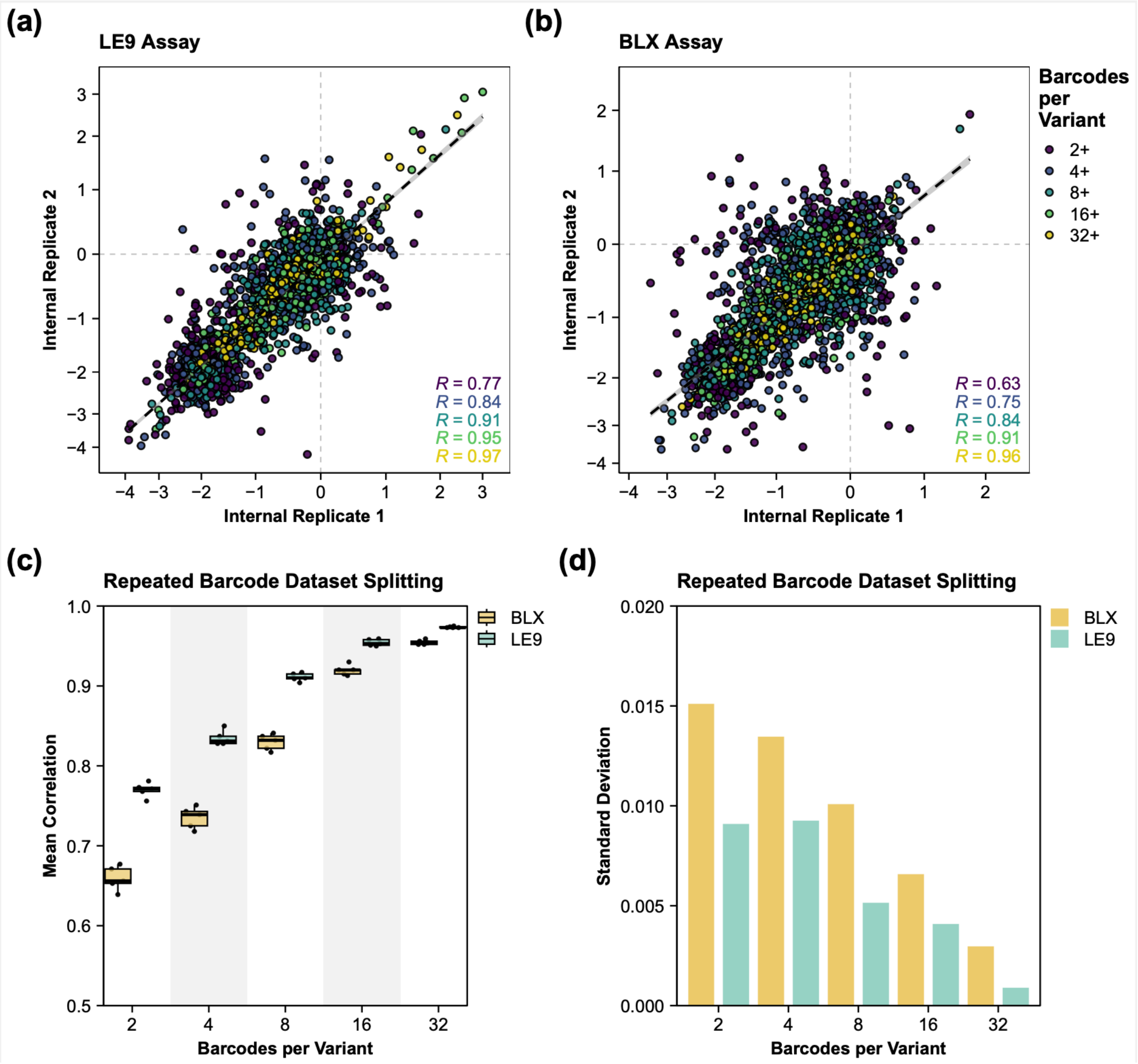

Appendix Figure S3. Analysis of internal replicates

(a-b) Correlation between internal replicates generated by randomly allocating half of the barcodes in each experiment to replicates 1 and 2, and filtering variants by the number of barcodes. R, Pearson correlations for LE9 (a) and BLX (b). (c) Pearson correlation between internal replicates generated as above; random allocation of barcodes to internal replicates was repeated 5 times. (d) Standard deviations of replicated correlation values from (c).

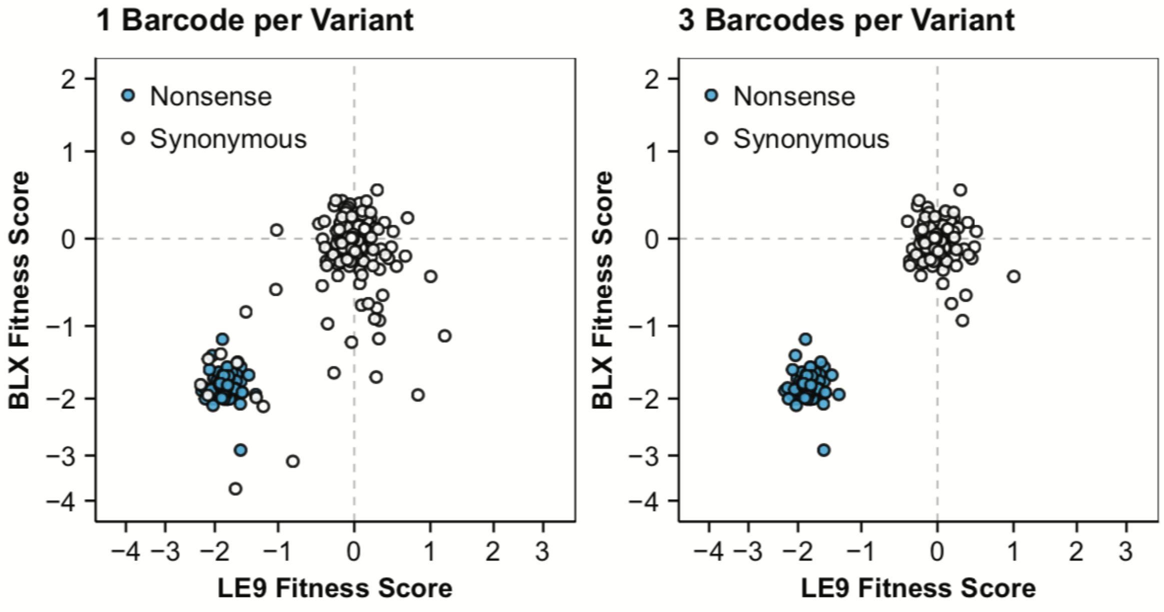

Appendix Figure S4. Noise in synonymous and nonsense variant scores as a function of barcode counts.

Comparison of variant fitness scores of synonymous and nonsense variants in the LE9 and BLX assays, filtered by number of barcodes per variant (one, left versus three, right).

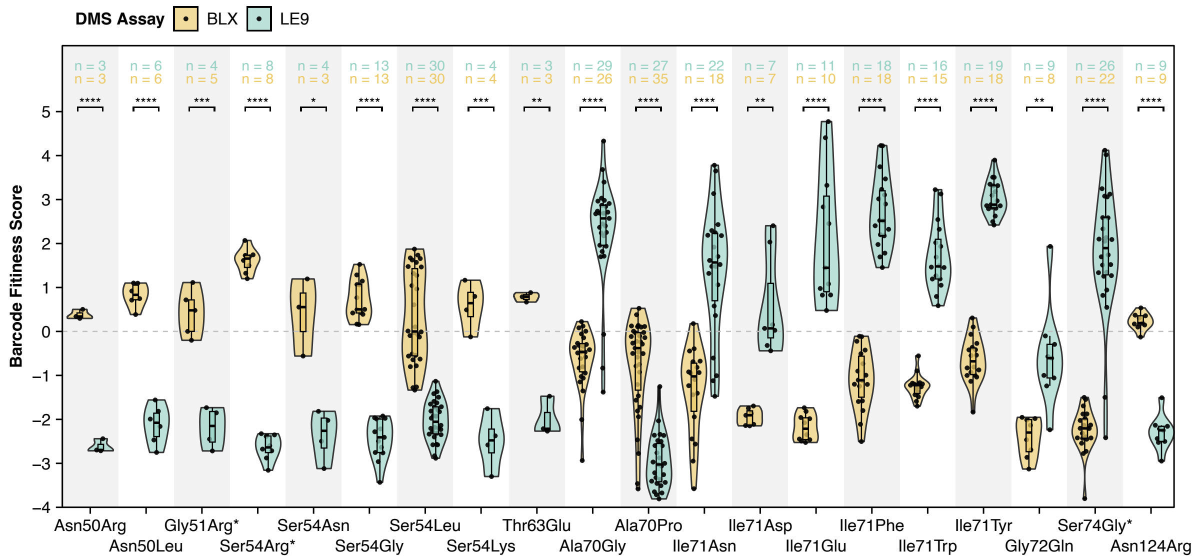

Appendix Figure S5. Variants with inverse changes in DNA-binding

The top 20 variants with the highest shifts in variant fitness score between the LE9 assay (teal) and BLX assay (yellow) are shown. Black dots represent individual barcodes for each variant. Asterisks (*) on the x-axis denote pathogenic variants identified in human patients. All p-values are calculated using Wilcoxon test and adjusted using FDR. *, p<=0.05; **, p<0.01; ***, p<0.001; ****, p<0.0001.

Row	Name	Sequence	Source
1	LE9 x 3	ATGAGAGATCTTTCCGCTCATTGCCCATTCAAATACAATTGTAGATCATGAGAGATCTTTCCGCTCATTGCCCATTCAAATACAATTGTAGATCATGAGAGATCTTTCCGCTCATTGCCCATTCAAATACAATTGTAGATC	Aota *et al* (2003)
2	SIMO x 1	GTGTCAATGCCTGAAGTGATACGCTCTGA	Bhatia *et al* (2013)
3	SIMO x 3	GTGTCAATGCCTGAAGTGATACGCTCTGAGTGTCAATGCCTGAAGTGATACGCTCTGAGTGTCAATGCCTGAAGTGATACGCTCTGA	Bhatia *et al* (2013)
4	N3 x 1	CTTTGTTATAGGCTAAGCTCTCAGTAATCCCAAACAAAAGAGT	Inoue *et al* (2007)
5	N3 x 3	CTTTGTTATAGGCTAAGCTCTCAGTAATCCCAAACAAAAGAGTCTTTGTTATAGGCTAAGCTCTCAGTAATCCCAAACAAAAGAGTCTTTGTTATAGGCTAAGCTCTCAGTAATCCCAAACAAAAGAGT	Inoue *et al* (2007)
6	DC5 x 1	AAATATTCATTGTTGTTGCTCACCTACCATGGATCC	Hayashi *et al* (1987)
7	DC5 x 3	AAATATTCATTGTTGTTGCTCACCTACCATGGATCCAAATATTCATTGTTGTTGCTCACCTACCATGGATCCAAATATTCATTGTTGTTGCTCACCTACCATGGATCC	Hayashi *et al* (1987)
8	DC5CON x 3	AAATATTCATTGTTGATGTTCACGCATCATGGATCCAAATATTCATTGTTGATGTTCACGCATCATGGATCCAAATATTCATTGTTGATGTTCACGCATCATGGATCC	Narasimhan *et al* (2015)
9	ACACA	AGGTTTGTATTCATTCTTTTCAGCTTGCTTGGATTTAGGTTTGTATTCATTCTTTTCAGCTTGCTTGGATTTAGGTTTGTATTCATTCTTTTCAGCTTGCTTGGATTT	Narasimhan *et al* (2015)
10	P6CON x 3	TTCAGGAAAAATTTTCACGCTTGAGTTCACAGCTCGAGTTTCAGGAAAAATTTTCACGCTTGAGTTCACAGCTCGAGTTTCAGGAAAAATTTTCACGCTTGAGTTCACAGCTCGAGT	Epstein *et al* (1994)
11	CRY x 3	ATTTCACGCATGAGTGCACATTTCACGCATGAGTGCACATTTCACGCATGAGTGCAC	Xu *et al* (1999)
12	TFAP2A	GGAAGAACTGCCTGTTCACAATTTTCACCATACTGACCATTCGTTTTAAACGATTTTTTTCTTTTCTTTTCTGTGCTCTGACAGTCTGTTTGACTCAGATGCAGTTGTTTTCAAATAACATGAACGGAGCCTAACTGTACATGTCCTTGGCATAATTGCAATGCACAGTGCAGCAGCAGGTGAAGCGAGGCATTGTCTGTCAAGACTGTTTTTGTTGGCTGCTTCTCAGATTGTGTGTGGGATTCTAACCTCGGGCACAA	Coutinho *et al* (2011)
13	FOXP2	TGCGACTGGTCATTTGTGTTTAGGCCGCATAACATGTGGCTGTATGGAATTTACAGTGCGCCTCCAGTAGTAACTCCATTTCCCATCGGCCTCTCCAGAGACCAGGCGAAATGTCAAGCCGTGGCTTTGAAAGAGAGATCGAGCGAGGAGGGGGAATAGAGAGAGAGAGAGAAAGGGACAGGGTGTTATATCTGCTGGTAACAAACACTTATGAACATTACTCATGCTTGGGTGACCAGAGAC	Coutinho *et al* (2011)
14	BLX x 3	CAGTCAAGCGTACAGTCAAGCGTACAGTCAAGCGTA	Unpublished Data, FitzPatrick Lab
15	P6 SELEX x 3	TGTGTTCACTCAAGCGGAAATGTGTTCACTCAAGCGGAAATGTGTTCACTCAAGCGGAAA	Unpublished Data, FitzPatrick Lab
16	FIMO x 3	CAGTCAGGCGTGCAGTCAGGCGTGCAGTCAGGCGTG	Unpublished Data, FitzPatrick Lab
17	XL	CCTCCTTGAAACTGAAATTTTAGCATGTGATTAATTAACTTGTAATATTCTAATCAAGCTTATAAAAGAGCACTGTTGGGCGTGAGTGGAGGCGCCGGAAAAAAGCATCGAAAAAACTTAGAAAA	Redden & Alper (2015)
18	Position 1 Mutagenesis primer	CTTGGCACAGCCGCCATGNNSAACAGCCACAGTGGAGTAAAC	This Study
19	Barcode_Amp_F	AATGATACGGCGACCACCGAGATCTACACNNNNNNACACTCTTTCCCTACACGACGCTCTTCCGATCTAGCGACAAGGAGGGCTGAGGACCGGTAGT	This Study
20	Barcode_Amp_R	CAAGCAGAAGACGGCATACGAGATNNNNNNGTGACTGGAGTTCAGACGTGTGCTCTTCCGATCTCTCGAGGCGCGCCATACTCA	This Study
21	Custom_Rd1_Seq	AGCGACAAGGAGGGCTGAGGACCGGTAGT	This Study

Appendix Table S1. Oligonucleotide sequences

Rows 1 to 16 correspond to the bait sequences used to screen for PAX6-mediated antibiotic resistance, using either single (x1) or triple tandem repeats (x3). Row 17 represents a synthetic minimal yeast promoter. Row 18 shows an example of a mutagenic oligonucleotide used to introduce all possible amino acids at position 2 in the PAX6 paired domain using a degenerate NNS sequence. Similar primers shifted at 3 nucleotide increments were used to cover all codons intended for mutagenesis in PAX6. Rows 19 to 20 depict the primers used to simultaneously amplify barcode DNA and append Illumina-compatible adapters. The 6N sequences correspond to unique dual indices used in demultiplexing during next-generation sequencing. Row 21 is the custom read 1 sequencing primer used in barcode sequence. N, any nucleotide; S, guanine or cytosine.

	AUROC	n_Pathogenic	n_Benign	optimal	path_cor	beni_cor	path_inc	beni_inc	accuracy	AUBPRC	ROC_score	BPR_score
DMS_BLX_+Geneticin_ABS	0.954	89	31	0.610	81	29	8	2	0.917	0.950	1.000	1.000
MetaRNN	0.932	91	31	0.978	77	29	14	2	0.869	0.923	0.966	0.948
MutPred	0.929	91	29	0.862	81	25	10	4	0.883	0.921	0.983	0.966
DMS_BLX_-Geneticin_ABS	0.926	89	31	1.014	81	27	8	4	0.900	0.922	0.947	0.930
DMS_LE9_-Geneticin_ABS	0.922	89	31	1.028	76	28	13	3	0.867	0.926	0.930	0.982
DMS_LE9_+Geneticin_ABS	0.913	89	31	1.443	77	29	12	2	0.883	0.903	0.912	0.860
MutationAssessor	0.901	91	31	3.070	82	24	9	7	0.869	0.902	0.897	0.897
DMS_BLX_-Geneticin	0.898	89	31	1.014	77	28	12	3	0.875	0.904	0.877	0.877
CONDEL	0.895	91	31	0.694	67	28	24	3	0.779	0.886	0.862	0.810
VESPAl	0.894	91	31	0.492	83	23	8	8	0.869	0.890	0.845	0.828
DMS_LE9_-Geneticin	0.892	89	31	0.977	73	28	16	3	0.842	0.903	0.807	0.842
DMS_BLX_+Geneticin	0.891	89	31	-0.610	78	29	11	2	0.892	0.908	0.789	0.912
MetaLR	0.889	91	31	0.989	66	29	25	2	0.779	0.856	0.793	0.690
ESM-1v	0.886	91	31	-12.026	86	23	5	8	0.893	0.852	0.759	0.672
ClinPred	0.886	91	31	0.991	84	23	7	8	0.877	0.823	0.741	0.621
DMS_LE9_+Geneticin	0.885	89	31	-1.443	76	29	13	2	0.875	0.885	0.772	0.789
CPT	0.883	91	31	0.735	78	24	13	7	0.836	0.869	0.707	0.759
DEOGEN2	0.878	91	31	0.969	80	24	11	7	0.852	0.857	0.707	0.707
BayesDel	0.876	91	31	0.479	74	27	17	4	0.828	0.803	0.690	0.586
DeepSAV	0.875	91	31	0.827	76	25	15	6	0.828	0.869	0.672	0.776
M-CAP	0.868	91	31	0.839	78	23	13	8	0.828	0.859	0.655	0.724
SIFT4G	0.868	37	23	0.025	36	19	1	4	0.917	0.795	0.561	0.439
VARITY_R	0.866	91	31	0.928	77	24	14	7	0.828	0.847	0.638	0.655
VARITY_ER	0.865	91	31	0.793	85	19	6	12	0.852	0.860	0.621	0.741
REVEL	0.858	91	31	0.908	82	23	9	8	0.861	0.825	0.603	0.638
FATHMM	0.846	91	31	-6.070	72	25	19	6	0.795	0.822	0.586	0.603
SIFT	0.825	10	16	0.030	8	12	2	4	0.769	0.769	0.433	0.356
EVE	0.813	91	31	0.935	71	25	20	6	0.787	0.746	0.552	0.466
DeepSequence	0.812	91	31	-7.092	88	19	3	12	0.877	0.750	0.534	0.466
Polyphen2_HumVar	0.802	91	30	0.999	54	26	37	4	0.661	0.762	0.500	0.534
VEST4	0.796	91	31	0.887	61	27	30	4	0.721	0.742	0.466	0.448
SuSPect	0.794	91	31	60.000	70	24	21	7	0.770	0.766	0.448	0.517
fathmm-XF	0.782	91	31	0.964	78	22	13	9	0.820	0.758	0.466	0.517
Eigen	0.782	91	31	6.180	87	18	4	13	0.861	0.704	0.414	0.362
MVP	0.775	91	31	0.971	86	16	5	15	0.836	0.681	0.414	0.345
Polyphen2_HumDiv	0.763	91	27	0.996	78	16	13	11	0.797	0.690	0.397	0.345
SNAP2	0.762	91	31	67.000	58	26	33	5	0.689	0.765	0.397	0.517
CADD	0.747	91	31	25.000	88	13	3	18	0.828	0.712	0.362	0.379
PonP2	0.746	91	31	0.841	69	21	22	10	0.738	0.716	0.328	0.379
fathmm-MKL	0.743	91	31	0.956	69	21	22	10	0.738	0.724	0.310	0.431
mutationTCN	0.742	91	20	-11.410	60	15	31	5	0.676	0.724	0.446	0.482
BLOSUM62	0.728	79	28	-1.000	66	15	13	13	0.757	0.660	0.298	0.246
PROVEAN	0.724	91	30	-4.010	74	20	17	10	0.777	0.631	0.293	0.259
LASSIE	0.710	64	29	0.001	48	20	16	9	0.731	0.721	0.246	0.298
Grantham	0.696	91	31	60.000	72	17	19	14	0.730	0.621	0.259	0.172
PonPS	0.691	91	31	0.410	74	17	17	14	0.746	0.631	0.241	0.241
phyloP	0.683	91	31	7.189	73	20	18	11	0.762	0.621	0.224	0.207
NetDiseaseSNP	0.655	91	31	0.440	75	15	16	16	0.738	0.622	0.190	0.207

sequence_unet	0.639	91	31	0.205	69	16	22	15	0.697	0.666	0.172	0.276
fitCons	0.633	91	31	0.598	83	11	8	20	0.770	0.578	0.155	0.155
DANN	0.624	91	31	0.996	69	17	22	14	0.705	0.551	0.147	0.121
MetaSVM	0.616	91	31	1.001	65	20	26	11	0.697	0.507	0.138	0.017
GenoCanyon	0.610	91	31	1.000	83	10	8	21	0.762	0.568	0.103	0.138
MutationTaster	0.581	91	31	1.000	88	6	3	25	0.770	0.545	0.086	0.103
SiPhy	0.540	91	31	13.716	80	10	11	21	0.738	0.513	0.069	0.086
PrimateAI	0.538	91	31	0.876	68	14	23	17	0.672	0.510	0.052	0.034
GERP++	0.524	91	31	3.550	91	6	0	25	0.795	0.511	0.034	0.069
phastCons	0.516	91	31	1.000	91	1	0	30	0.754	0.508	0.017	0.017
LRT	0.450	51	19	0.000	10	18	41	1	0.400	0.525	0.000	0.054

Appendix Table S2. Summary of variant classification by VEPs and DMS

Estimation of the ability of DMS assay data and VEPs to accurately classify pathogenic and benign PAX6 variants are shown as a measure of the area under the receiver operating characteristic curve (ROC AUC). Area under the precision-recall curve (AUBPRC) was also calculated. Normalised to the best performer, these are summarised as ROC_score and BPR_score, respectively. For accuracy and optimal thresholds determinations, see methods.

**VEP or score**	**Classification**	**Data source**	**Reference**
SIFT	Unsupervised	Run locally, scripts available from: https://sift.bii.a-star.edu.sg/www/code.html	Sim *et al* (2012)
SIFT4G	Unsupervised	dbNSFP 4.2	Vaser *et al* (2016)
phyloP	Unsupervised	dbNSFP 4.2	Pollard *et al* (2010)
BLOSUM62	Unsupervised	https://www.ncbi.nlm.nih.gov/Class/FieldGuide/BLOSUM62.txt	Henikoff & Henikoff (1992)
LRT	Unsupervised	dbNSFP 4.2	Chun & Fay (2009)
SiPHy	Unsupervised	dbNSFP 4.2	Garber *et al* (2009)
GERP++	Unsupervised	dbNSFP 4.2	Davydov *et al* (2010)
Grantham	Unsupervised	Available from referenced paper	Grantham (1974)
PROVEAN	Unsupervised	dbNSFP 4.2	Choi *et al* (2012)
MutationAssessor	Unsupervised	dbNSFP 4.2	Reva *et al* (2011)
DeepSequence	Unsupervised	Run locally, scripts available from: https://github.com/debbiemarkslab/DeepSequence	Riesselman *et al* (2018)
GenoCanyon	Unsupervised	dbNSFP 4.2	Lu *et al* (2015)
EVE	Unsupervised	Downloaded from https://evemodel.org/	Frazer *et al* (2021)
mutationTCN	Unsupervised	http://mtban.kaist.ac.kr/	Kim & Kim (2020)
VESPAl	Unsupervised	https://zenodo.org/record/5905863	Marquet *et al* (2022)
ESM-1v	Unsupervised (no fine-tuning)	Run locally, scripts available from: https://github.com/facebookresearch/esm	Meier *et al* (2021)
phastCons	Unsupervised	dbNSFP 4.2	Siepel & Haussler (2005)
fitCons	Unsupervised	dbNSFP 4.2	Gulko *et al* (2015)
Polyphen-2 (HumVar and HumDiv models)	Supervised	dbNSFP 4.2	Adzhubei *et al* (2010)
Eigen	Supervised	dbNSFP 4.2	Ionita-Laza *et al* (2016)
SuSPect	Supervised	http://www.sbg.bio.ic.ac.uk/suspect/index.html	Yates *et al* (2014)
DEOGEN2	Supervised	dbNSFP 4.2	Raimondi *et al* (2017)
VEST4	Supervised	dbNSFP 4.2	Carter *et al*

			(2013)
SNAP2	Supervised	https://rostlab.org/services/snap2web/	Hecht *et al* (2015)
MutPred	Supervised	dbNSFP 4.2	Pejaver *et al* (2020)
PonP2	Supervised	http://structure.bmc.lu.se/PON-P2/	Niroula *et al* (2015)
PonPS	Supervised	http://structure.bmc.lu.se/PON-PS/	Niroula & Vihinen (2017)
Fathmm-XF	Supervised	dbNSFP 4.2	Rogers *et al* (2018)
Fathmm	Supervised	dbNSFP 4.2	Shihab *et al* (2013)
Fathmm-MKL	Supervised	dbNSFP 4.2	Shihab *et al* (2015)
PrimateAI	Supervised	dbNSFP 4.2	Sundaram *et al* (2018)
VARITY (R and ER models)	Supervised	http://varity.varianteffect.org/	Wu *et al* (2021)
REVEL	Supervised	dbNSFP 4.2	Ioannidis *et al* (2016)
CONDEL	Supervised	http://bbglab.irbbarcelona.org/fannsdb/	González-Pérez & López-Bigas (2011)
MetaLR	Supervised	dbNSFP 4.2	Dong *et al* (2015)
MetaSVM	Supervised	dbNSFP 4.2	Dong *et al* (2015)
M-CAP	Supervised	dbNSFP 4.2	Jagadeesh *et al* (2016)
MVP	Supervised	dbNSFP 4.2	Qi *et al* (2021)
CADD	Supervised	dbNSFP 4.2	Kircher *et al* (2014)
DANN	Supervised	dbNSFP 4.2	Quang *et al* (2015)
MetaRNN	Supervised	dbNSFP 4.2	Li *et al* (2022)
ClinPred	Supervised	dbNSFP 4.2	Alirezaie *et al* (2018)
BayesDel	Supervised	dbNSFP 4.2	Feng (2017)
CPT	Supervised	https://zenodo.org/records/7954657	Jagota *et al* (2023)
DeepSAV	Supervised	http://prodata.swmed.edu/DBSAV/	Pei & Grishin (2021)
LASSIE	Unsupervised	http://compgen.cshl.edu/LASSIE/ (genome browser track)	Huang & Siepel (2019)
NetDiseaseSNP	Supervised	http://www.cbs.dtu.dk/services/NetDiseaseSNP/ (now defunct)	Johansen *et al* (2013)
Sequence_unet	Supervised	Run locally, scripts available from	Dunham *et al*

		https://github.com/allydunham/sequence_unet	(2023)
MutationTaster	Supervised	dbNSFP 4.2	Schwarz *et al* (2010)

Appendix Table S3. List of Variant Effect Predictors

List of variant effect predictors used in this study with classification (supervised versus unsupervised), data source and literature references.

APPENDIX REFERENCES

Adzhubei IA, Schmidt S, Peshkin L, Ramensky VE, Gerasimova A, Bork P, Kondrashov AS & Sunyaev SR (2010) A method and server for predicting damaging missense mutations. *Nat Methods* 7: 248–249

Alirezaie N, Kernohan KD, Hartley T, Majewski J & Hocking TD (2018) ClinPred: Prediction Tool to Identify Disease-Relevant Nonsynonymous Single-Nucleotide Variants. *Am J Hum Genet* 103: 474–483

Aota S, Nakajima N, Sakamoto R, Watanabe S, Ibaraki N & Okazaki K (2003) Pax6 autoregulation mediated by direct interaction of Pax6 protein with the head surface ectoderm-specific enhancer of the mouse Pax6 gene. *Dev Biol* 257: 1–13

Bhatia S, Bengani H, Fish M, Brown A, Divizia MT, de Marco R, Damante G, Grainger R, van Heyningen V & Kleinjan DA (2013) Disruption of autoregulatory feedback by a mutation in a remote, ultraconserved PAX6 enhancer causes aniridia. *Am J Hum Genet* 93: 1126–34

Carter H, Douville C, Stenson PD, Cooper DN & Karchin R (2013) Identifying Mendelian disease genes with the variant effect scoring tool. *BMC Genomics* 14 Suppl 3: S3

Choi Y, Sims GE, Murphy S, Miller JR & Chan AP (2012) Predicting the functional effect of amino acid substitutions and indels. *PloS One* 7: e46688

Chun S & Fay JC (2009) Identification of deleterious mutations within three human genomes. *Genome Res* 19: 1553–1561

Coutinho P, Pavlou S, Bhatia S, Chalmers KJ, Kleinjan DA & van Heyningen V (2011) Discovery and assessment of conserved Pax6 target genes and enhancers. *Genome Res* 21: 1349–59

Davydov EV, Goode DL, Sirota M, Cooper GM, Sidow A & Batzoglou S (2010) Identifying a High Fraction of the Human Genome to be under Selective Constraint Using GERP++. *PLOS Comput Biol* 6: e1001025

Dong C, Wei P, Jian X, Gibbs R, Boerwinkle E, Wang K & Liu X (2015) Comparison and integration of deleteriousness prediction methods for nonsynonymous SNVs in whole exome sequencing studies. *Hum Mol Genet* 24: 2125–2137

Dunham AS, Beltrao P & AlQuraishi M (2023) High-throughput deep learning variant effect prediction with Sequence UNET. *Genome Biol* 24: 110

Epstein J, Cai J, Glaser T, Jepeal L & Maas R (1994) Identification of a Pax paired domain recognition sequence and evidence for DNA-dependent conformational changes. *J Biol Chem* 269: 8355–8361

Feng B-J (2017) PERCH: A Unified Framework for Disease Gene Prioritization. *Hum Mutat* 38: 243–251

Frazer J, Notin P, Dias M, Gomez A, Min JK, Brock K, Gal Y & Marks DS (2021) Disease variant prediction with deep generative models of evolutionary data. *Nature* 599: 91–95

Garber M, Guttman M, Clamp M, Zody MC, Friedman N & Xie X (2009) Identifying novel constrained elements by exploiting biased substitution patterns. *Bioinformatics* 25: i54

González-Pérez A & López-Bigas N (2011) Improving the Assessment of the Outcome of Nonsynonymous SNVs with a Consensus Deleteriousness Score, Condel. *Am J Hum Genet* 88: 440–449

Grantham R (1974) Amino acid difference formula to help explain protein evolution. *Science* 185: 862–864

Gulko B, Hubisz MJ, Gronau I & Siepel A (2015) A method for calculating probabilities of fitness consequences for point mutations across the human genome. *Nat Genet* 47: 276–283

Hayashi S, Goto K, Okada TS & Kondoh H (1987) Lens-specific enhancer in the third intron regulates expression of the chicken delta 1-crystallin gene. *Genes Dev* 1: 818–828

Hecht M, Bromberg Y & Rost B (2015) Better prediction of functional effects for sequence variants. *BMC Genomics* 16: S1

Henikoff S & Henikoff JG (1992) Amino acid substitution matrices from protein blocks. *Proc Natl Acad Sci U S A* 89: 10915

Huang Y-F & Siepel A (2019) Estimation of allele-specific fitness effects across human protein-coding sequences and implications for disease. *Genome Res* 29: 1310–1321

Inoue M, Kamachi Y, Matsunami H, Imada K, Uchikawa M & Kondoh H (2007) PAX6 and SOX2-dependent regulation of the Sox2 enhancer N-3 involved in embryonic visual system development. *Genes Cells* 12: 1049–1061

Ioannidis NM, Rothstein JH, Pejaver V, Middha S, McDonnell SK, Baheti S, Musolf A, Li Q, Holzinger E, Karyadi D, *et al* (2016) REVEL: An Ensemble Method for Predicting the Pathogenicity of Rare Missense Variants. *Am J Hum Genet* 99: 877–885

Ionita-Laza I, McCallum K, Xu B & Buxbaum JD (2016) A spectral approach integrating functional genomic annotations for coding and noncoding variants. *Nat Genet* 48: 214–220

Jagadeesh KA, Wenger AM, Berger MJ, Guturu H, Stenson PD, Cooper DN, Bernstein JA & Bejerano G (2016) M-CAP eliminates a majority of variants of uncertain significance in clinical exomes at high sensitivity. *Nat Genet* 48: 1581–1586

Jagota M, Ye C, Albors C, Rastogi R, Koehl A, Ioannidis N & Song YS (2023) Cross-protein transfer learning substantially improves disease variant prediction. *Genome Biol* 24: 182

Johansen MB, Izarzugaza JMG, Brunak S, Petersen TN & Gupta R (2013) Prediction of Disease Causing Non-Synonymous SNPs by the Artificial Neural Network Predictor NetDiseaseSNP. *PLOS ONE* 8: e68370

Kim HY & Kim D (2020) Prediction of mutation effects using a deep temporal convolutional network. *Bioinformatics* 36: 2047–2052

Kircher M, Witten DM, Jain P, O'Roak BJ, Cooper GM & Shendure J (2014) A general framework for estimating the relative pathogenicity of human genetic variants. *Nat Genet* 46: 310–315

Li C, Zhi D, Wang K & Liu X (2022) MetaRNN: differentiating rare pathogenic and rare benign missense SNVs and InDels using deep learning. *Genome Med* 14: 115

Lu Q, Hu Y, Sun J, Cheng Y, Cheung K-H & Zhao H (2015) A Statistical Framework to Predict Functional Non-Coding Regions in the Human Genome Through Integrated Analysis of Annotation Data. *Sci Rep* 5: 10576

Marquet C, Heinzinger M, Olenyi T, Dallago C, Erckert K, Bernhofer M, Nechaev D & Rost B (2022) Embeddings from protein language models predict conservation and variant effects. *Hum Genet* 141: 1629–1647

Meier J, Rao R, Verkuil R, Liu J, Sercu T & Rives A (2021) Language models enable zero-shot prediction of the effects of mutations on protein function. *bioRxiv*: 450648 doi:10.1101/2021.07.09.450648 [PREPRINT]

Narasimhan K, Pillay S, Huang YH, Jayabal S, Udayasuryan B, Veerapandian V, Kolatkar P, Cojocaru V, Pervushin K & Jauch R (2015) DNA-mediated cooperativity facilitates the co-selection of cryptic enhancer sequences by SOX2 and PAX6 transcription factors. *Nucleic Acids Res* 43: 1513

Niroula A, Urolagin S & Vihinen M (2015) PON-P2: Prediction Method for Fast and Reliable Identification of Harmful Variants. *PLoS ONE* 10: e0117380

Niroula A & Vihinen M (2017) Predicting Severity of Disease-Causing Variants.

Pei J & Grishin NV (2021) The DBSAV Database: Predicting Deleteriousness of Single Amino Acid Variations in the Human Proteome. *J Mol Biol* 433: 166915

Pejaver V, Urresti J, Lugo-Martinez J, Pagel KA, Lin GN, Nam H-J, Mort M, Cooper DN, Sebat J, Iakoucheva LM, *et al* (2020) Inferring the molecular and phenotypic impact of amino acid variants with MutPred2. *Nat Commun* 11: 5918

Pollard KS, Hubisz MJ, Rosenbloom KR & Siepel A (2010) Detection of nonneutral substitution rates on mammalian phylogenies. *Genome Res* 20: 110–121

Qi H, Zhang H, Zhao Y, Chen C, Long JJ, Chung WK, Guan Y & Shen Y (2021) MVP predicts the pathogenicity of missense variants by deep learning. *Nat Commun* 12: 510

Quang D, Chen Y & Xie X (2015) DANN: a deep learning approach for annotating the pathogenicity of genetic variants. *Bioinforma Oxf Engl* 31: 761–763

Raimondi D, Tanyalcin I, Ferté J, Gazzo A, Orlando G, Lenaerts T, Rooman M & Vranken W (2017) DEOGEN2: prediction and interactive visualization of single amino acid variant deleteriousness in human proteins. *Nucleic Acids Res* 45: W201–W206

Redden H & Alper HS (2015) The development and characterization of synthetic minimal yeast promoters. *Nat Commun* 6

Reva B, Antipin Y & Sander C (2011) Predicting the functional impact of protein mutations: application to cancer genomics. *Nucleic Acids Res* 39: e118

Riesselman AJ, Ingraham JB & Marks DS (2018) Deep generative models of genetic variation capture the effects of mutations. *Nat Methods* 15: 816–822

Rogers MF, Shihab HA, Mort M, Cooper DN, Gaunt TR & Campbell C (2018) FATHMM-XF: accurate prediction of pathogenic point mutations via extended features. *Bioinformatics* 34: 511–513

Schwarz JM, Rödelsperger C, Schuelke M & Seelow D (2010) MutationTaster evaluates disease-causing potential of sequence alterations. *Nat Methods* 7: 575–576

Shihab HA, Gough J, Cooper DN, Stenson PD, Barker GLA, Edwards KJ, Day INM & Gaunt TR (2013) Predicting the functional, molecular, and phenotypic consequences of amino acid substitutions using hidden Markov models. *Hum Mutat* 34: 57–65

Shihab HA, Rogers MF, Gough J, Mort M, Cooper DN, Day INM, Gaunt TR & Campbell C (2015) An integrative approach to predicting the functional effects of non-coding and coding sequence variation. *Bioinforma Oxf Engl* 31: 1536–1543

Siepel A & Haussler D (2005) Phylogenetic Hidden Markov Models. In *Statistical Methods in Molecular Evolution*, Nielsen R (ed) pp 325–351. New York, NY: Springer

Sim N-L, Kumar P, Hu J, Henikoff S, Schneider G & Ng PC (2012) SIFT web server: predicting effects of amino acid substitutions on proteins. *Nucleic Acids Res* 40: W452-457

Sundaram L, Gao H, Padigepati SR, McRae JF, Li Y, Kosmicki JA, Fritzilas N, Hakenberg J, Dutta A, Shon J, *et al* (2018) Predicting the clinical impact of human mutation with deep neural networks. *Nat Genet* 50: 1161–1170

Vaser R, Adusumalli S, Leng SN, Sikic M & Ng PC (2016) SIFT missense predictions for genomes. *Nat Protoc* 11: 1–9

Williamson KA, Hall HN, Owen LJ, Livesey BJ, Hanson IM, Adams GGW, Bodek S, Calvas P, Castle B, Clarke M, *et al* (2019) Recurrent heterozygous PAX6 missense variants cause severe bilateral microphthalmia via predictable effects on DNA–protein interaction. *Genet Med 2019 223* 22: 598–609

Wu Y, Li R, Sun S, Weile J & Roth FP (2021) Improved pathogenicity prediction for rare human missense variants. *Am J Hum Genet* 108: 1891–1906

Xu HE, Rould MA, Xu W, Epstein JA, Maas RL & Pabo CO (1999) Crystal structure of the human Pax6 paired domain-DNA complex reveals specific roles for the linker region and carboxy-terminal subdomain in DNA binding. *Genes Dev* 13: 1263–75

Yates CM, Filippis I, Kelley LA & Sternberg MJE (2014) SuSPect: Enhanced Prediction of Single Amino Acid Variant (SAV) Phenotype Using Network Features. *J Mol Biol* 426: 2692–2701
