# Supplementary material for: Deep mutational scanning quantifies DNA binding and predicts clinical outcomes of PAX6 variants
Source: Mol Syst Biol. 2024 Jun 7;20(7):825–44. doi: 10.1038/s44320-024-00043-8 (PMC11219921; doi:10.1038/s44320-024-00043-8)
Supplement: Supplementary file 8 — Appendix Figure 1A Source Data [file 44320_2024_43_MOESM8_ESM.pptx]

## Slide 1
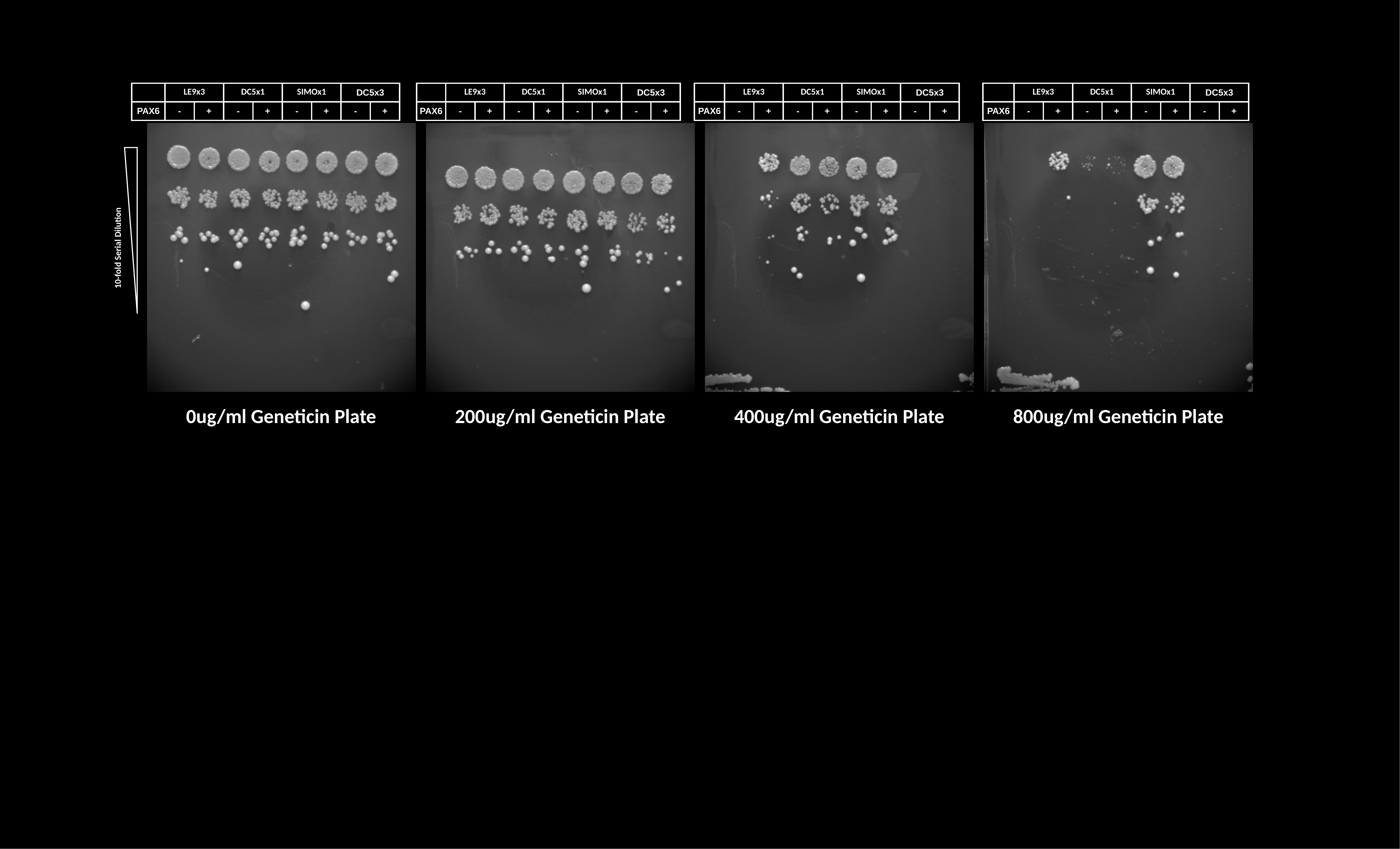

| | LE9x3 | | DC5x1 | | SIMOx1 | | DC5x3 | |
| --- | --- | --- | --- | --- | --- | --- | --- | --- |
| PAX6 | - | + | - | + | - | + | - | + |
| | LE9x3 | | DC5x1 | | SIMOx1 | | DC5x3 | |
| --- | --- | --- | --- | --- | --- | --- | --- | --- |
| PAX6 | - | + | - | + | - | + | - | + |
| | LE9x3 | | DC5x1 | | SIMOx1 | | DC5x3 | |
| --- | --- | --- | --- | --- | --- | --- | --- | --- |
| PAX6 | - | + | - | + | - | + | - | + |
| | LE9x3 | | DC5x1 | | SIMOx1 | | DC5x3 | |
| --- | --- | --- | --- | --- | --- | --- | --- | --- |
| PAX6 | - | + | - | + | - | + | - | + |
10-fold Serial Dilution
0ug/ml Geneticin Plate
200ug/ml Geneticin Plate
400ug/ml Geneticin Plate
800ug/ml Geneticin Plate

## Slide 2
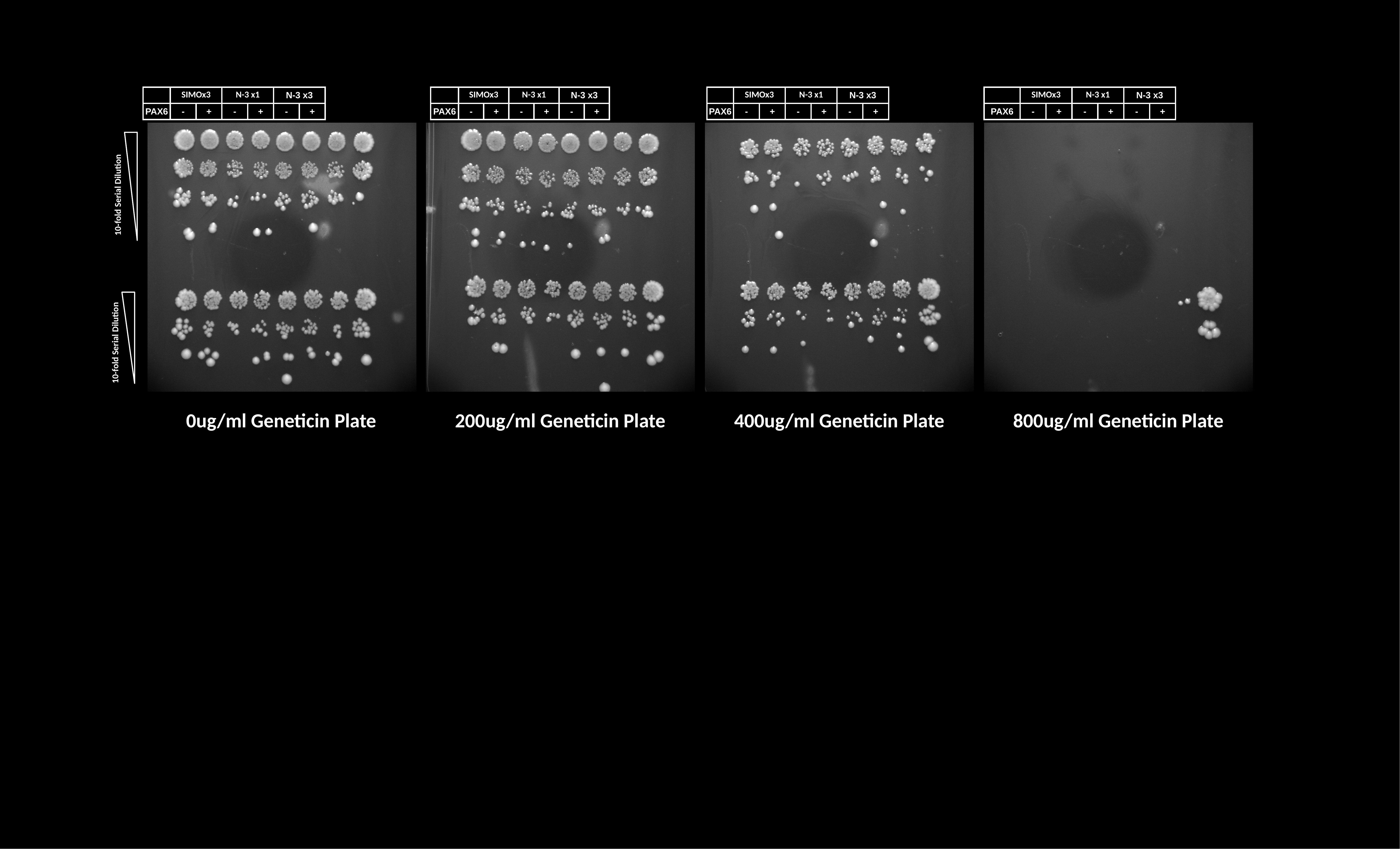

| | SIMOx3 | | N-3 x1 | | N-3 x3 | |
| --- | --- | --- | --- | --- | --- | --- |
| PAX6 | - | + | - | + | - | + |
| | SIMOx3 | | N-3 x1 | | N-3 x3 | |
| --- | --- | --- | --- | --- | --- | --- |
| PAX6 | - | + | - | + | - | + |
| | SIMOx3 | | N-3 x1 | | N-3 x3 | |
| --- | --- | --- | --- | --- | --- | --- |
| PAX6 | - | + | - | + | - | + |
| | SIMOx3 | | N-3 x1 | | N-3 x3 | |
| --- | --- | --- | --- | --- | --- | --- |
| PAX6 | - | + | - | + | - | + |
10-fold Serial Dilution
10-fold Serial Dilution
0ug/ml Geneticin Plate
200ug/ml Geneticin Plate
400ug/ml Geneticin Plate
800ug/ml Geneticin Plate

## Slide 3
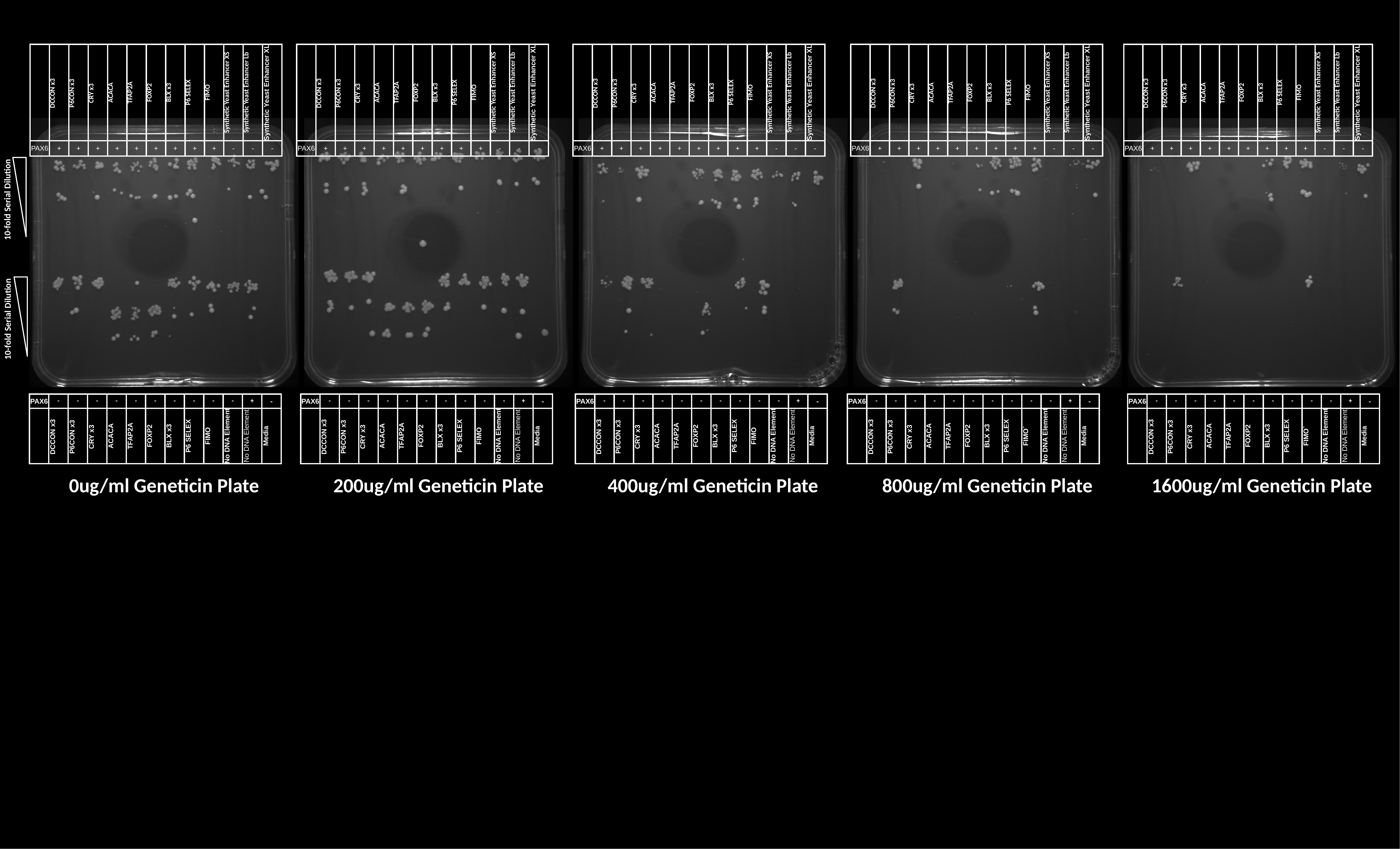

| | DCCON x3 | P6CON x3 | CRY x3 | ACACA | TFAP2A | FOXP2 | BLX x3 | P6 SELEX | FIMO | Synthetic Yeast Enhancer XS | Synthetic Yeast Enhancer Lb | Synthetic Yeast Enhancer XL |
| --- | --- | --- | --- | --- | --- | --- | --- | --- | --- | --- | --- | --- |
| PAX6 | + | + | + | + | + | + | + | + | + | - | - | - |
| | DCCON x3 | P6CON x3 | CRY x3 | ACACA | TFAP2A | FOXP2 | BLX x3 | P6 SELEX | FIMO | Synthetic Yeast Enhancer XS | Synthetic Yeast Enhancer Lb | Synthetic Yeast Enhancer XL |
| --- | --- | --- | --- | --- | --- | --- | --- | --- | --- | --- | --- | --- |
| PAX6 | + | + | + | + | + | + | + | + | + | - | - | - |
| | DCCON x3 | P6CON x3 | CRY x3 | ACACA | TFAP2A | FOXP2 | BLX x3 | P6 SELEX | FIMO | Synthetic Yeast Enhancer XS | Synthetic Yeast Enhancer Lb | Synthetic Yeast Enhancer XL |
| --- | --- | --- | --- | --- | --- | --- | --- | --- | --- | --- | --- | --- |
| PAX6 | + | + | + | + | + | + | + | + | + | - | - | - |
| | DCCON x3 | P6CON x3 | CRY x3 | ACACA | TFAP2A | FOXP2 | BLX x3 | P6 SELEX | FIMO | Synthetic Yeast Enhancer XS | Synthetic Yeast Enhancer Lb | Synthetic Yeast Enhancer XL |
| --- | --- | --- | --- | --- | --- | --- | --- | --- | --- | --- | --- | --- |
| PAX6 | + | + | + | + | + | + | + | + | + | - | - | - |
| | DCCON x3 | P6CON x3 | CRY x3 | ACACA | TFAP2A | FOXP2 | BLX x3 | P6 SELEX | FIMO | Synthetic Yeast Enhancer XS | Synthetic Yeast Enhancer Lb | Synthetic Yeast Enhancer XL |
| --- | --- | --- | --- | --- | --- | --- | --- | --- | --- | --- | --- | --- |
| PAX6 | + | + | + | + | + | + | + | + | + | - | - | - |
10-fold Serial Dilution
10-fold Serial Dilution
| PAX6 | - | - | - | - | - | - | - | - | - | - | + | - |
| --- | --- | --- | --- | --- | --- | --- | --- | --- | --- | --- | --- | --- |
| | DCCON x3 | P6CON x3 | CRY x3 | ACACA | TFAP2A | FOXP2 | BLX x3 | P6 SELEX | FIMO | No DNA Element | No DNA Element | Media |
| PAX6 | - | - | - | - | - | - | - | - | - | - | + | - |
| --- | --- | --- | --- | --- | --- | --- | --- | --- | --- | --- | --- | --- |
| | DCCON x3 | P6CON x3 | CRY x3 | ACACA | TFAP2A | FOXP2 | BLX x3 | P6 SELEX | FIMO | No DNA Element | No DNA Element | Media |
| PAX6 | - | - | - | - | - | - | - | - | - | - | + | - |
| --- | --- | --- | --- | --- | --- | --- | --- | --- | --- | --- | --- | --- |
| | DCCON x3 | P6CON x3 | CRY x3 | ACACA | TFAP2A | FOXP2 | BLX x3 | P6 SELEX | FIMO | No DNA Element | No DNA Element | Media |
| PAX6 | - | - | - | - | - | - | - | - | - | - | + | - |
| --- | --- | --- | --- | --- | --- | --- | --- | --- | --- | --- | --- | --- |
| | DCCON x3 | P6CON x3 | CRY x3 | ACACA | TFAP2A | FOXP2 | BLX x3 | P6 SELEX | FIMO` | No DNA Element | No DNA Element | Media |
| PAX6 | - | - | - | - | - | - | - | - | - | - | + | - |
| --- | --- | --- | --- | --- | --- | --- | --- | --- | --- | --- | --- | --- |
| | DCCON x3 | P6CON x3 | CRY x3 | ACACA | TFAP2A | FOXP2 | BLX x3 | P6 SELEX | FIMO` | No DNA Element | No DNA Element | Media |
0ug/ml Geneticin Plate
200ug/ml Geneticin Plate
400ug/ml Geneticin Plate
800ug/ml Geneticin Plate
1600ug/ml Geneticin Plate
