# Supplementary material for: Deep mutational scanning quantifies DNA binding and predicts clinical outcomes of PAX6 variants
Source: Mol Syst Biol. 2024 Jun 7;20(7):825–44. doi: 10.1038/s44320-024-00043-8 (PMC11219921; doi:10.1038/s44320-024-00043-8)
Supplement: Supplementary file 9 — Expanded View Figures [file 44320_2024_43_MOESM9_ESM.pdf]

Expanded View Figures

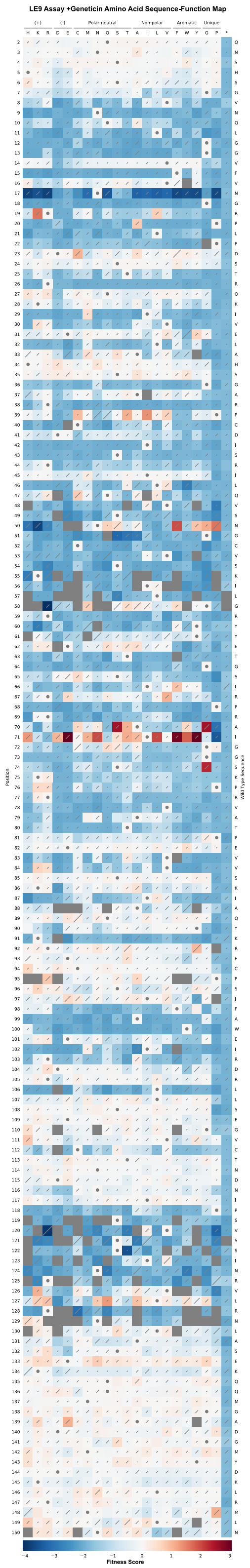

Figure EV1. Variant effect map of PAX6 paired domain in LE9 strain with geneticin treatment.

Colours and symbols as in Fig. 2. Source data are available online for this figure.

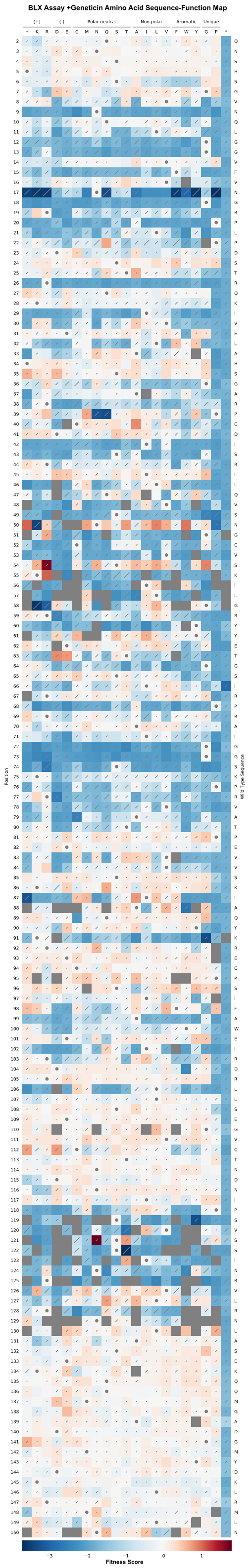

Figure EV2. Variant effect map of PAX6 paired domain in BLX strain with geneticin treatment.

Colours and symbols as in Fig. 2.

 © The Author(s)

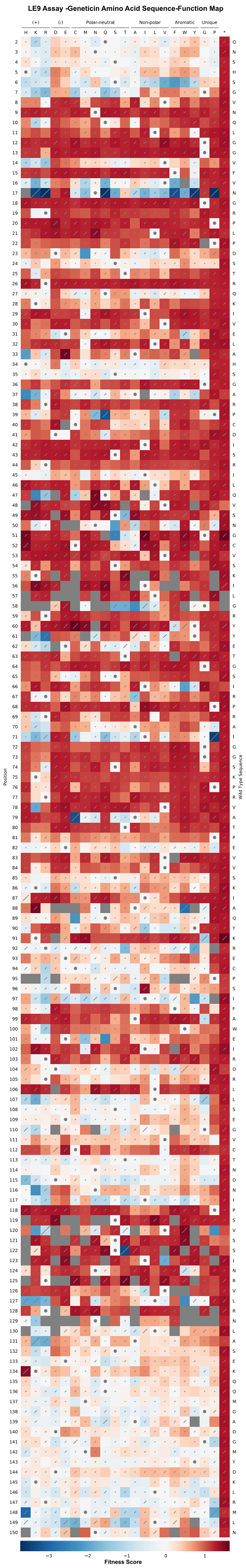

Figure EV3. Variant effect map of PAX6 paired domain in LE9 strain without geneticin.

Colours and symbols as in Fig. 2.

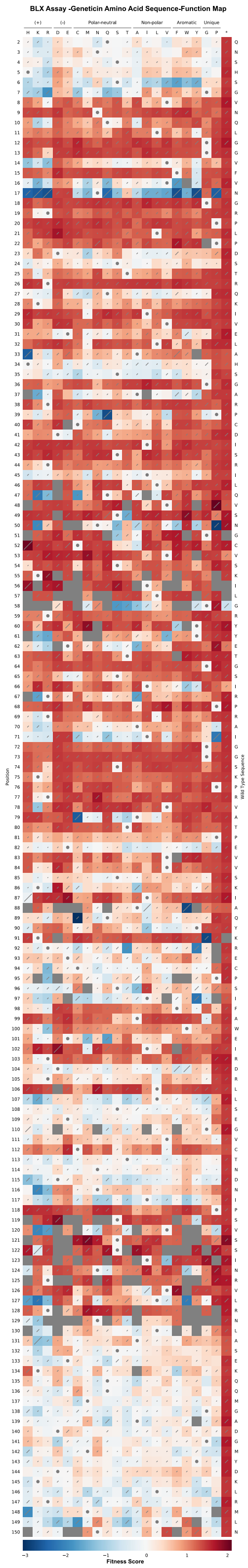

Figure EV4. Variant effect map of PAX6 paired domain in BLX strain without geneticin.

Colours and symbols as in Fig. 2.

 © The Author(s)
